# Use of Intravaginal Cooling to Provide Symptom Relief in Women With Vulvovaginal Candidiasis and Reduce Immunopathology in an Accompanying Mouse Model

**DOI:** 10.1093/infdis/jiaf028

**Published:** 2025-01-13

**Authors:** Junko Yano, Kimberly Langdon, Michael Swor, Mairi C Noverr, Paul L Fidel

**Affiliations:** Department of Oral and Craniofacial Biology, School of Dentistry, LSU Health, New Orleans, Louisiana, USA; Coologics, Inc., Cleveland, Ohio, USA; Physicians Care Clinical Research Center, Sarasota Memorial Hospital, Sarasota, Florida, USA; Department of Oral and Craniofacial Biology, School of Dentistry, LSU Health, New Orleans, Louisiana, USA; Department of Oral and Craniofacial Biology, School of Dentistry, LSU Health, New Orleans, Louisiana, USA

**Keywords:** vulvovaginal candidiasis, *Candida albicans*, inflammation, hyphae, non-pharmacologic therapies

## Abstract

**Background:**

Vulvovaginal candidiasis (VVC), caused primarily by *Candida albicans*, is treated with anti-fungal drugs, often with variable efficacy and relapses. New therapeutic strategies, including drug-free alternatives, are needed. Upon overgrowth or environmental triggers, *C. albicans* commensal yeast transitions into hyphae resulting in an aberrant immunopathologic neutrophil response that contributes to the characteristic signs and symptoms of vaginitis. The purpose of this study was to evaluate the efficacy of an intravaginal cooling device (Vlisse) in women with VVC to provide symptom relief via putative reversal of *C. albicans* hyphae to yeast, with additional proof of principle in an animal model.

**Methods:**

Five women with VVC were instructed to use the device twice daily for 3 days. Vulvovaginal symptoms were monitored and scored for each use, followed by pelvic examination at 30 days. A mouse model of VVC employed cooled micro stir rods to evaluate the cooling effect on fungal morphology and vaginal immunopathology.

**Results:**

Clinical cure was achieved in all women. In the mouse model, the insertion of pre-cooled magnetic rods intravaginally for short periods over 3 days, reduced the immunopathogenic neutrophil infiltration and hyphae.

**Conclusions:**

Intravaginal cooling provides clinical cure for VVC and proof of principle in an animal model.

## Teaser

Current therapies for vulvovaginal candidiasis often fail to prevent recurrences. This study demonstrates intravaginal cooling as an effective, nonpharmacologic approach to reduce symptoms by reverting *Candida albicans* hyphae to yeast, validated through pilot clinical trials and in proof-of-concept animal studies.

Vulvovaginal candidiasis (VVC), predominantly caused by *Candida albicans*, is a common fungal infection, affecting approximately 75% of women at least once during their reproductive years [1]. An additional 5%–8% experience recurrent VVC (RVVC), defined as ≥3 episodes per year [2–4]. VVC is characterized by acute vaginal inflammation in response to fungal overgrowth within the vaginal cavity [5], with symptoms including itching, burning, swelling, erythema, excoriation, pain and discharge [6, 7]. While current topical or oral antifungal therapies provide short-term relief by suppressing fungal burden, they do not offer complete cure, leading to frequent relapses. Since RVVC can occur without known predisposing factors (such as hormonal changes, microbiome imbalances, uncontrolled diabetes mellitus), treatment often necessitates maintenance antifungal therapies [3, 8]. VVC and RVVC negatively affect the quality of life and increase healthcare burden for otherwise healthy women worldwide.

A key pathogenic factor of *C. albicans* is its ability to transition from yeast to hyphal morphology, which is considered a hallmark virulence factor for VVC [9, 10]. Clinical and animal studies have demonstrated that this yeast-to-hyphal transition activates an immunopathogenic response by polymorphonuclear neutrophils (PMNs) in the vaginal lumen [11–13]. Despite accumulating evidence of their roles in VVC/RVVC immunopathogenesis, there are no therapies designed to specifically revert *C. albicans* hyphae to yeast or reduce PMN infiltration. Such strategies may offer a favorable outcome by reverting *C. albicans* to its harmless yeast form and restoring vaginal homeostasis without the use of antifungal drugs.

Triggers for *C. albicans* hyphal transition include elevated temperature (>37°C) and pH (>5.5) [14–16]. Hence, one strategy to revert *C. albicans* from the pathogenic hyphal form to the commensal yeast form is to cool its growth environment, thereby reducing triggers for the immunopathogenic response and alleviating vaginal symptoms. While the yeast form may once again switch to pathogenic hyphae and trigger more episodes, the cooling strategy could offer a fast-acting, nonpharmacologic alternative to drugs that can be slower acting, costly, and promote resistance.

Coologics has developed and patented Vlisse, a reusable device designed to maintain a cooled vaginal environment below homeostatic temperature. Similar to a tampon, it is intended to fit securely against the vaginal wall and allows smooth insertion and removal by users.

The focus of this pilot clinical study was to evaluate the efficacy of intravaginal cooling treatment by the Vlisse device in reducing VVC symptoms. In addition, proof of principle was assessed using a similar concept and treatment regimen employing cooled micro stir rods in an established mouse model of VVC with the ability to evaluate hyphal presence and PMN infiltration.

## METHODS

### Clinical Trial Participants and Enrollment

Ten women with physician-diagnosed VVC were initially screened for eligibility into the pilot clinical study at the Physicians Clinical Care Research clinic in Sarasota, Florida, in accordance with the Institutional Review Board at the Sarasota Memorial Hospital. Five patients were formally enrolled into the study based on eligibility/exclusion criteria (Supplementary Table 1). The other 5 women were excluded due to potential confounding conditions, including uncontrolled diabetes (n = 2), concurrent urinary tract infection (n = 1), concurrent bacterial vaginosis (n = 1), and intolerance to intravaginal products (n = 1). VVC symptoms were classified as described elsewhere, with modifications [17, 18]. Briefly, vulvovaginal signs and symptoms (itching, burning, pain, dyspareunia, swelling, redness and discharge) were scored on a 0–4 severity scale: 0, none; 1, minimal; 2, mild; 3, moderate; 4, severe. Composite symptom scores were calculated by summing individual scores across all symptom categories at baseline and each subsequent self-assessment, with scores ≥7 indicating moderate-to-severe VVC. Vaginal swab samples were examined by means of wet-mount microscopy for the detection of *C. albicans* using the potassium hydroxide (KOH; 10%) test. Pelvic examination and vaginal swab collection were conducted at enrollment and follow-up visits 30 days after conclusion of treatments. Clinical cure was defined by meeting both of the following criteria: (1) complete resolution of all signs and symptoms [18–21], and (2) a negative KOH test result at 30-day follow-up.

### Intravaginal Cooling Device and Treatment Regimens

The vaginal cooling device (Vlisse; Coologics) (Supplementary Figure 1) is a reusable device consisting of a medical-grade ethyl vinyl acetate shell prefilled with a proprietary hydrogel optimized for cold temperature retention and freeze-thaw stability. The device is tampon shaped, allowing for a secure fit against the vaginal wall and smooth insertion and removal by the users. After baseline assessment of VVC signs and symptoms, participants were provided with the device, instructions for use, and a diary to record self-assessment of each sign/symptom category using the scoring system (Supplementary Table 1) at the time of each pretreatment and post-treatment episode. The standard treatment regimen consisted of 30-minute vaginal cooling sessions twice daily, approximately 12 hours apart, for 3 days. Patients also received rescue medication to be taken if necessary (fluconazole [Diflucan; Pfizer]; 150 mg, taken orally). A follow-up pelvic examination was conducted 30 days after the final treatment.

### Animal Studies

#### Mice

Female C3H mice (5–7 weeks old) were purchased from Charles River Laboratories and housed in AAALAC-approved facilities in the LSU Health School of Dentistry. All animal protocols were approved by the Institutional Animal Care and Use Committee of LSU Heath, New Orleans, Louisiana. Following an acclimation period of one week, mice were subcutaneously injected with 0.1 mg of β-estradiol-17-valerate (Sigma) in 100 µL of sesame oil. Injections continued weekly throughout the experimental period.

#### Microorganisms

*C. albicans* 96113 (American Type Culture Collection), a vaginal isolate, was maintained in a 20% glycerol stock medium at −80°C. A 10-µL aliquot from the glycerol stock was streaked onto Sabouraud dextrose agar (BD diagnostics) and cultured at 35°C for 48 hours. A single colony was transferred into 10 mL of yeast extract-peptone-dextrose broth and incubated at 30°C for 18 hours with shaking at 200 rpm until reaching the stationary phase. Following incubation, *C. albicans* cells were washed 3 times in sterile phosphate-buffered saline (PBS) and enumerated using a hemocytometer.

#### Intravaginal Cooling Treatment

Estrogen-treated, inoculated or uninoculated mice were briefly restrained to expose the vaginal opening [22]. A sterile magnetic micro stir rod (ThermoFisher, Supplementary Figure 2), precooled to −20°C, was inserted into the vagina using fine-tipped forceps. These magnetic rods, coated with inert, Food and Drug Administration–grade polytetrafluoroethylene (Teflon) polymer, were securely positioned along the full length of the vagina but remained visible at the vaginal opening. Mice remained unanesthetized during the procedure, were immediately released into their cages, and resumed normal activity without signs of distress. Control groups included mice treated with identical, noncooled rods or left untreated. To minimize confounding effects of insertion/removal on intravaginal temperature, control rods were maintained at 35°C (the baseline intravaginal temperature) and tested in parallel. In each treatment group, rods were replaced with fresh, temperature-conditioned rods every 10 minutes for 40 minutes, for a series of 4 individual applications per treatment. This procedure was repeated twice daily for 3 days starting at 2 days after inoculation.

#### Intravaginal Temperature

Temperature of the vaginal cavity was measured using a digital thermometer installed with a 19-mm-length rectal probe (±0.1°C precision; ThermoWorks). Temperature measurements were taken at baseline and after treatment and recorded longitudinally for each animal.

#### Mouse Model of VVC

Intravaginal inoculation of *C. albicans* was conducted as described elsewhere [22]. Briefly, 3 days after estrogen injection, mice were administered 20 µL of PBS containing *C. albicans* 96115 (5 × 10^4^) into the vaginal lumen. At specific time points after inoculation, vaginal lavage was performed under isoflurane anesthesia. Briefly, 100 µL PBS was introduced into the vaginal lumen and aspirated several times with gentle agitation, using a pipette tip. Resulting lavage fluids were transferred individually into microcentrifuge tubes, and 10-µL aliquots from each lavage sample were removed to determine fungal burden, hyphal scoring and PMN quantification. Groups of 3–5 mice were evaluated longitudinally throughout the post-inoculation period.

#### Vaginal Fungal Burden

Serial dilutions of the lavage fluid were cultured on Sabouraud-dextrose agar plates supplemented with gentamycin (Invitrogen). After incubation for 24 hours at 37°C, colonies were enumerated and were expressed as colony-forming units per microliter.

#### Hyphal Scoring

Aliquots of vaginal lavage fluid were observed using wet-mount microscopy at 400× magnification to identify *C. albicans* morphology. Hyphal presence was scored on a 0–4 scale: 0, none; +1, sparse hyphae; +2, small amounts of hyphae in several fields; +3, large amounts of hyphae in several fields; and +4, masses of hyphae in most fields.

#### PMN Quantification

Smears of 10 µL of vaginal lavage fluid were stained using the Papanicolaou technique. PMNs, identified by their trilobed nuclei, were the predominant infiltrating leukocytes, as reported elsewhere [12]. PMNs were enumerated in 5 nonadjacent fields per sample at 400× magnification, and the mean values were used for data analyses.

## RESULTS

### Pilot Clinical Trial for Treatment of Symptomatic VVC With an Intravaginal Cooling Device

Five women with symptomatic VVC were formally enrolled and given the Vlisse device (Supplementary Figure 1), along with instructions for use and a treatment schedule, consisting of 30-minute vaginal cooling sessions, twice daily for 3 days, approximately 12 hours apart. Preliminary vaginal temperature measurements conducted in 3 healthy women without VVC indicated a steep reduction from 35.9°C ± 1.27°C to 24.2 ± 5.83°C during the first 10 minutes after insertion, followed by a gradual return to baseline within 30–40 minutes, with no significant variation in temperature shifts among participants (analysis of variance [ANOVA]; *P* = .95) (Supplementary Figure 3*A*).

**Figure 1.**
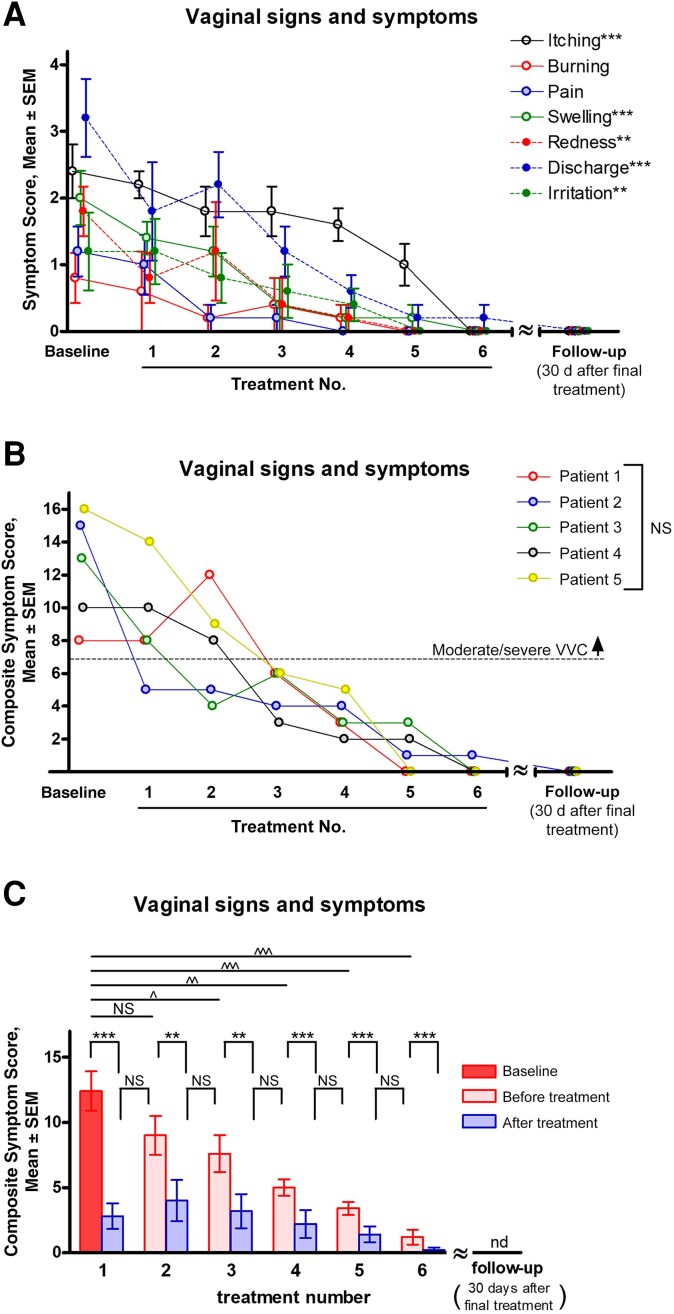
Effects of intravaginal cooling treatment in women with moderate to severe vulvovaginal candidiasis (VVC). Five women were enrolled in the clinical trial study based on eligibility/exclusion criteria. For assessment of vulvovaginal signs and symptoms (itching, burning, pain, dysuria, swelling, redness and discharge) at baseline and subsequent time points, a scoring system was used for quantitative evaluation on a scale of 0–4 as a measure of the severity of vaginitis: 0, none; 1, minimal; 2, mild; 3, moderate; or 4, severe. The standard treatment regimen consisted of 30-minute vaginal cooling sessions twice daily, approximately 12 hours apart, for 3 days, totaling 6 individual treatment sessions. The participants self-assessed and recorded their vaginal signs and symptoms before and after treatment for each use, followed by a follow-up pelvic examination conducted 30 days after treatment. *A*, Scores per symptom category from baseline to after treatment assessed at each use. *B*, Composite symptom scores per participant from baseline to after treatment assessed at each use. Horizontal dotted line represents the threshold for composite symptom scores (≥7), defined as moderate to severe VVC. *C*, Composite symptom scores per use at baseline and before and after treatment. Dot plot (*A*), bar heights (*C*) and error bars (*A* and *C*) reflect the mean ± SEM of the values computed from the 5 patients. Data were analyzed using the repeated-measures analysis of variance (ANOVA) comparing change over successive treatments (*A*), the 2-way ANOVA followed by the Bonferroni posthoc test comparisons between patients (*B*), or the 1-way ANOVA followed by the unpaired Student *t* test comparing the baseline/pretreatment and each posttreatment value (**P < .01; ***P < .001), comparing the posttreatment and the next pretreatment interval between treatments (^##^*P* < .01; ^###^*P* < .001), or comparing the baseline and each pretreatment value (*^P* < .05; ^^*P* < .01; ^^^*P* < .001) (*C*). Abbreviations: ND, not detected; NS, not significant.

All participants reported a steady reduction in all symptom categories after the 3-day treatment regimen, with a total of 6 applications (Table 1). At baseline, itching, swelling and discharge were the most prominent symptoms and were significantly reduced after the 3-day treatment (*P* < .001). Redness and irritation also decreased significantly (*P* < .01), with mild pain and burning experienced only at baseline (Figure 1*A*).

**Table 1.** Symptom Assessment of Vulvovaginal Candidiasis Before and After Vaginal Cooling Treatment

Patient No. (Age, y)	Before or After Treatment	Composite VVC Symptom Score by Treatment No.						KOH Test Result^a^	
		1	2	3	4	5	6		
1 (38)	Before	8	8	12	6	3	0	Baseline	+
	After	6	6	8	6	2	0	F/U	−
2 (24)	Before	15	5	5	4	4	1	Baseline	+
	After	4	2	4	3	3	1	F/U	−
3 (38)	Before	12	8	4	6	3	3	Baseline	+
	After	1	0	1	1	0	0	F/U	−
4 (30)	Before	10	10	8	3	2	2	Baseline	+
	After	2	3	1	0	0	0	F/U	−
5 (38)	Before	16	14	9	6	5	0	Baseline	+
	After	1	9	2	1	2	0	F/U	−

Abbreviations: F/U, 30-day follow-up; KOH, potassium hydroxide; VVC, vulvovaginal candidiasis.

^a^A positive KOH test result (+) is an indication of *Candida albicans* hyphae.

All participants noted a similar declining trend (ANOVA; *P* = .93) in composite symptom scores falling below the threshold for moderate to severe VVC after 3 uses, followed by complete symptom relief at 30-day follow-up (Figure 1*B*). Specifically, symptom scores showed a significant reduction immediately after each treatment (*P* < .001 after the first, fourth, fifth and sixth treatments; *P* < .01 after the second and third treatments). A rebound in symptoms 12 hours after treatment was only observed after the first use (*P* < .01), followed by a consistent decline during subsequent treatments (ANOVA; *P* < .001) with no significant differences between any posttreatment assessment and the next pretreatment assessment. Significant differences from baseline were observed at each posttreatment assessment after the first treatment, whereas no significant differences were observed between all posttreatment assessments (ANOVA; *P* = .19) (Figure 1*C*). In all cases, symptom reduction was accompanied by the absence of detectable hyphae based on KOH tests at 30-day follow-up, indicating clinical cure (Table 1). None of the women required the rescue antifungal medication during the study period.

### Animal Studies

#### Implementation of Intravaginal Cooling Device in Mice

Building on the pilot trial, the established mouse model of VVC was modified to incorporate a similar intravaginal device suitable for animals. For this, magnetic micro stir rods with a smooth cylindrical structure, precooled to −20°C, were used to simulate the human device (Supplementary Figure 2). Initial experiments using a single insertion per mouse showed no significant change in intravaginal temperature between baseline and 30 minutes after treatment (34.95 ± 0.15°C vs 33.00 ± 0.70°C, respectively, *P* = .11). To enhance the cooling effect, rods were replaced with a new set of precooled rods every 10 minutes for 40 minutes, totaling 4 individual applications per mouse. Longitudinal temperature measurements indicated a constant decline in intravaginal temperature during the initial 20 minutes applications, achieving a significant reduction compared with the control groups (*P* < .01) and maintaining a stable local temperature at approximately 29°C until the end of the treatment (Supplementary Figure 3*B*). No visible signs of adverse reactions or trauma to mouse vaginal tissues after repeat applications were observed. To assess potential tissue damage, a toxicity analysis of vaginal lavage fluids showed no significant difference in lactate dehydrogenase (LDH) release in mice treated with cold or warmed rods (ANOVA; *P* = .50) (Supplementary Figure 3*C*).

#### Reduction of Hyphal Presence

**Figure 2.**
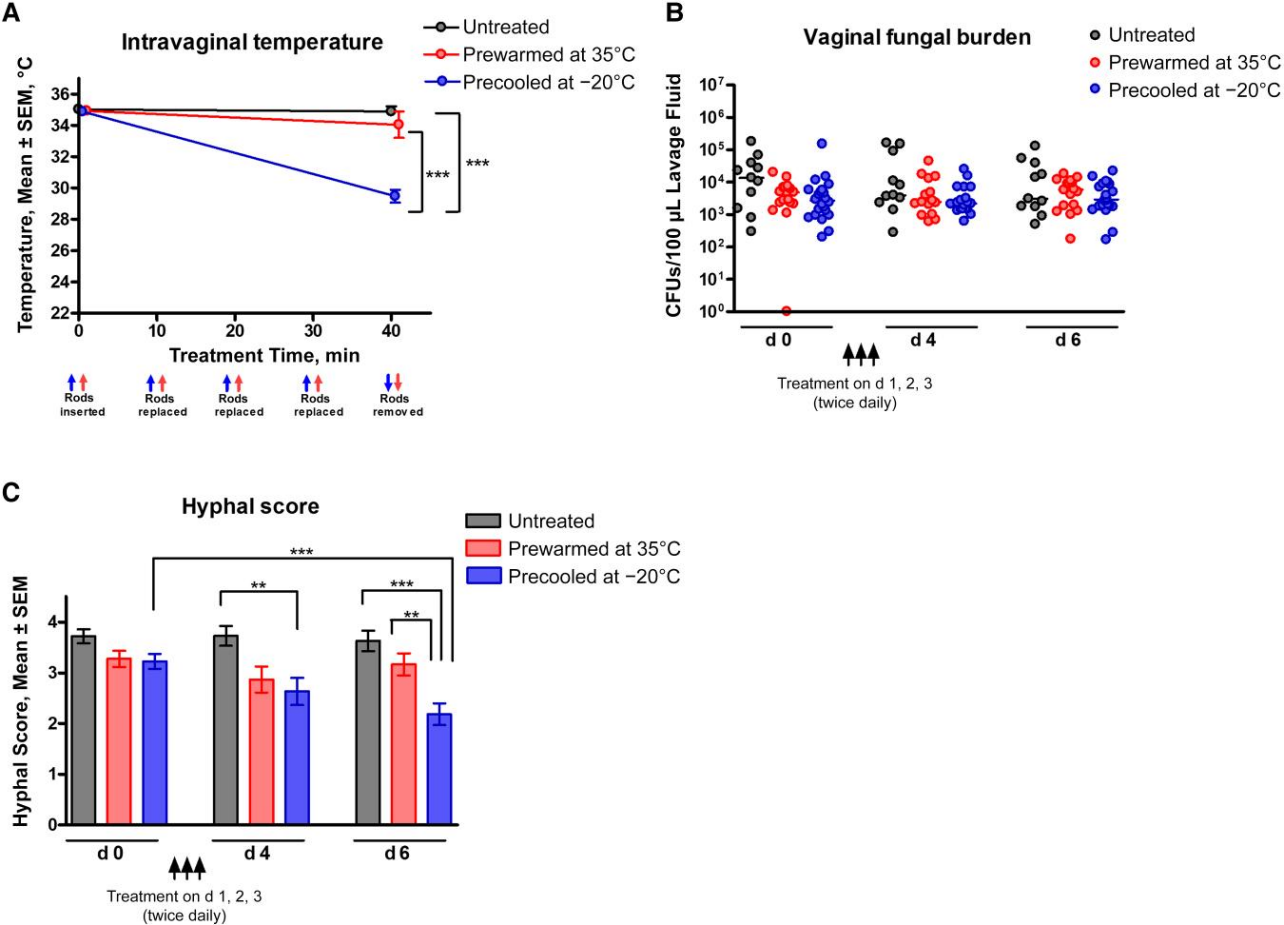
Effects of cooling treatment on presence of *Candida albicans* hyphae during experimental vaginitis. Estrogen-treated mice were intravaginally inoculated with *C. albicans* 96113. Four days after inoculation, mice were treated intravaginally with sterile magnetic micro rods prewarmed to 35°C or precooled to −20°C for a period of 40 minutes total (4 successive 10-minute treatment applications), twice daily for 3 days; mice in the control group remained untreated. Vaginal lavage fluid was collected on day 0 (2 days after inoculation, before treatment) and 1–3 days after the final treatment, on days 4 and 6 of the study period. *A*, Temperature shifts in the vaginal cavities of inoculated mice before and after treatment. *B*, Vaginal fungal burden before (day 0) and after (days 4–6) treatment, assessed by quantitative plate counts. *C*, Quantification of *C. albicans* hyphal scores by means of wet mount microscopy of the vaginal lavage samples, using the following scoring system; 0, no hyphae; 1, sparse hyphae; 2, small amounts of hyphae present in several fields; 3, large amounts of hyphae in several fields; or 4, masses of hyphae in most fields. *A, C,* Lines (*A*), bar heights (*C*) and error bars reflect group means ± SEM of the values computed from independent replicates of each of 4 unique sets of animals, with 2–6 mice per group. *B,* Dot plots represent data points for unique animals from 5 independent experiments, each including 2–6 mice per group. Data were analyzed using 2-way ANOVA comparing repeated measures between the 3 treatment groups followed by Bonferroni posthoc tests at the end point (*A*), Kruskal-Wallis test between groups at each time point (*B*), or unpaired Student *t* test to compare among groups at each time point or between each group at different time points (*C*). **P < .01; ***P < .001. Abbreviation: CFUs, colony-forming units.

To determine the effects of intravaginal cooling treatment on *C. albicans* filamentation, inoculated mice were subjected to the murine-optimized regimen and evaluated for vaginal fungal burden and levels of *C. albicans* hyphal presence. Similar to the preliminary temperature readings (Supplementary Figure 3*B*), the cooling treatment in inoculated mice resulted in a significant reduction in intravaginal temperature compared with mice receiving control treatment using rods prewarmed to 35°C or the untreated control (*P* < .001) (Figure 2*A*). After receiving the treatment twice daily for 3 days, all groups exhibited similar levels of vaginal fungal burden on days 4 and 6 (Figure 2*B*). Despite the constant fungal burden, assessment of fungal morphology by wet-mount microscopy revealed a significant reduction in *C. albicans* hyphae in vaginal lavage fluid from mice in the cooling treatment group compared with pretreatment levels (*P* < .001 for day 6), untreated controls (*P* < .01 for day 4 and *P* < .001 for day 6), and control treatments (*P* < .01 for day 6) (Figure 2*C*). A repeated measures analysis indicated a strong trend of successive reduction in hyphal scores in the cooling treatment group (*P* < .01), whereas no significant changes were observed in the control treatment and untreated groups (*P* = .89 and *P* = .58, respectively).

#### Reduction of VVC-Associated Inflammatory Response

**Figure 3.**
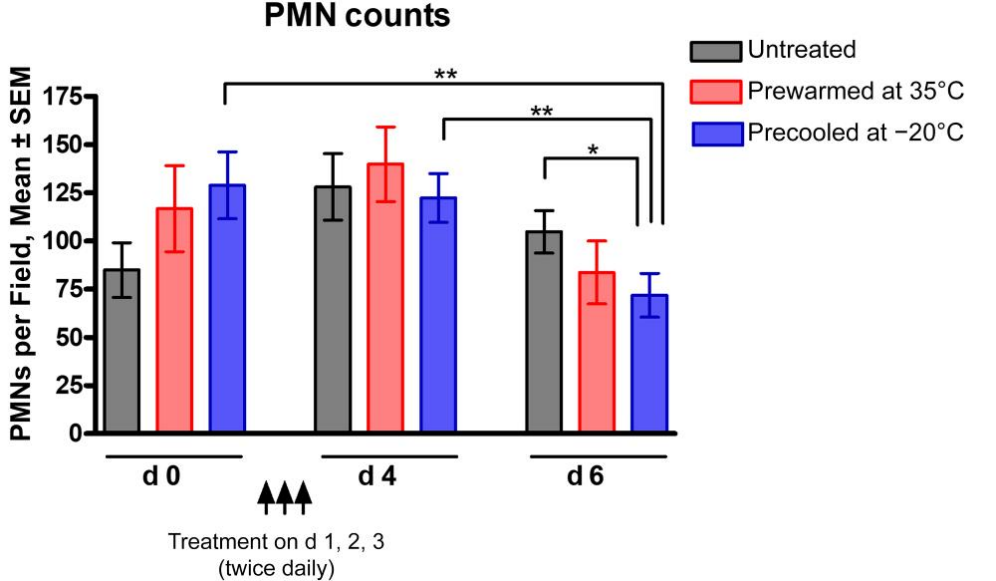
Effects of cooling treatment on vaginal inflammation during experimental vaginitis. Estrogen-treated mice were intravaginally inoculated with *Candida albicans* 96113. Two days after inoculation, mice either remained untreated or were treated intravaginally with sterile magnetic rods prewarmed to 35°C or precooled to −20°C for a treatment period of 40 minutes total (4 successive applications of 10-minute treatment), twice daily for 3 days. Vaginal lavage fluid was collected on day 0 (2 days after inoculation; before treatment) and 1–3 days after the final treatment, on days 4 and 6 of the study period. Vaginal cellular infiltrates were stained using the Papanicolaou technique and examined using light microscopy at ×400 magnification. The number of polymorphonuclear neutrophils (PMNs) was quantified in 5 nonadjacent fields per sample and averaged. Bar heights and error bars reflect the group mean ± SEM of the values computed from independent replicates of each of 5 unique sets of animals, with 2–6 mice per group. Data were analyzed using 1-way ANOVA, followed by Tukey posthoc tests and the Student *t* test to compare groups at each time point or between each group at different time points, respectively. *P < .05; **P < .01.

To further evaluate the effects of intravaginal cooling treatment on symptom outcomes, levels of PMN infiltrate, a hallmark indicator of symptomatic VVC, were assessed microscopically using Papanicolaou technique preparations of vaginal lavage fluid from mice subjected to the cooling treatment. Results from PMN quantification showed a significant reduction in PMN counts in vaginal lavage fluid on day 6, compared with pretreatment levels (*P* < .01), day 4 (*P* < .01), and the untreated control (*P* < .05) (Figure 3). Similar to the reduction in hyphal presence, a repeated-measures analysis indicated a significant regression in PMN migration after cooling treatment (*P* <.001), whereas the control treatment and untreated groups showed no statistical difference in PMN counts over the observation period (both *P* = .11).

## DISCUSSION

Despite the long-standing use of antifungal drugs for VVC and RVVC, current therapies often fail to provide complete cure or long-term relief. This shortcoming has led to considerable challenges for affected women, including increased healthcare burdens and reduced quality of life. This study had 2 main objectives: (1) to conduct a pilot clinical trial using the Vlisse device to treat VVC and (2) to establish proof of principle for the cooling therapeutic strategy in an established animal model of VVC.

Morphologic transition of *C. albicans* from yeast to hyphae is central to the pathogenicity in VVC. Studies have shown that hyphal formation triggers an aberrant inflammatory response by PMNs infiltrating the vaginal lumen [10] but with little effect on clearance of *Candida,* as heparan sulfate in the vaginal environment inhibits interactions between PMNs and *C. albicans* [23, 24]. This dysfunction results in persistent hyphal overgrowth and inflammation, which are key indicators of symptomatic VVC in both women and mouse models [11, 12, 25].

To this end, we proposed the concept of intravaginal cooling as a therapeutic strategy by reversing the morphology of *C. albicans* from pathogenic hyphae to commensal yeast. Below physiologic temperatures, *C. albicans* remains in the yeast form due to suppressed morphogenic gene expression even in the presence of potent hyphal inducers [15, 26, 27]. By targeting the morphologic state, cooling therapy aims to offer multifold benefits, including reducing the pathogenic fungal state, alleviating vaginal inflammation, and providing a nonpharmacologic, convenient alternative for managing VVC symptoms.

In this pilot clinical trial, 5 women with moderate to severe VVC underwent a regimen of 30-minute twice-daily intravaginal cooling using the Vlisse device. This approach demonstrated excellent compliance, with no adverse reactions reported. Notably, the treatment provided immediate relief, likely due first to the direct cooling effect on inflamed tissue rather than an instant reduction in hyphae, as symptoms rebounded within 12 hours of the first use. Conversely, sustained symptom relief observed after the second use suggests progressive reversion of hyphae to yeast over successive treatments. Overall, statistical tests applied between symptom scores at any posttreatment assessment compared with the following pretreatment assessment indicated that ≥2 treatments are needed for lasting effects. Although the small sample size and reliance on self-reported symptom measures are limitations, follow-up pelvic examinations and negative KOH test results 30 days after treatment confirmed clinical cure, extending symptom-free periods beyond the typical test-of-cure time frames (10–28 days) reported in previous antifungal drug studies for VVC treatment [19–21, 28].

While the results are promising, more rigorous trials with appropriate control groups will be necessary to validate the relief characteristics and long-term efficacy of this approach. Indeed, the prominence of discharge as a noted symptom does not align with a previous survey of >280 women, where discharge was reported in only about 50% of cases [6]. Nonetheless, the symptom categories chosen for VVC diagnosis and self-monitoring are considered appropriate and support the reliability of these data. Although use of cold compresses has been documented as complementary remedies for vulvovaginal discomfort [29–32], the mechanisms behind VVC symptom relief achieved by cooling remain unclear. Potential mechanisms include modulation of inflammation and reduction of hyphal-associated virulence factors through up-regulation of farnesol, a fungal quorum-sensing molecule. Farnesol is known for inhibiting yeast-to-hyphae transition, exhibiting immunomodulatory properties, and enhancing PMN antifungal activity [33–36].

Future studies aim to correlate symptom reduction with direct evidence of hyphae-to-yeast reversion during treatment to better elucidate these mechanisms. Another limitation of intravaginal cooling is its broad effect on the vaginal environment. It is certainly possible that vaginal cooling may affect not only *C. albicans* but also the broader vaginal microbiota. Despite being nonspecific to fungi, cooling is expected to produce an acute cold shock rather than a long-term reduction in local temperature. Cooling in this manner likely has more impact on *C. albicans* due to its morphologic plasticity. Although bacteria could also experience cold shock and possibly enter a brief suspended state, their high adaptability to cold environments suggests rapid recovery, if affected at all, since they do not undergo dynamic morphogenesis like fungi [37, 38]. Therefore, cooling is expected to have a minimal impact on the normal flora while effectively targeting the more susceptible *C. albicans*, leading to favorable outcomes.

Besides the pilot trial group, an additional 21 women with clinically or self-diagnosed VVC have used the Vlisse device in several investigator-initiated studies outside the formal trial (data not shown). While the treatment regimens varied, all women reported rapid symptom relief, with no additional treatment required for ≥30 days. Notably, in one study, fungal burden measured by cultures and quantitative polymerase chain reaction revealed that most women remained positive for *C. albicans* after treatment, despite achieving complete symptom resolution (ie, clinical cure), suggesting a shift from symptomatic infection to asymptomatic colonization. Together, these results support intravaginal cooling as a viable nonpharmacologic treatment alternative for both treating acute episodes and potentially preventing recurrences, warranting larger-scale clinical trials.

Building on these clinical data, a series of experiments were conducted in a mouse model of VVC, using a similar treatment approach compatible with mice. After testing various materials and configurations to simulate the Vlisse device, magnetic micro stir rods precooled to −20°C, applied 4 times with a freshly cooled rod every 10 minutes, achieved the optimal cooling effect in mouse vaginas. This protocol reduced intravaginal temperature to approximately 29°C for 30 minutes without epithelial damage. This process was performed twice daily for 3 days, mimicking the clinical treatment regimen.

Results from inoculated mice indicated significant reductions in hyphal scores and PMN migration 24–48 hours after treatment. Sustained vaginal fungal burden across the groups was expected due to the nonfungicidal nature of the treatment that was only meant to provoke hyphae-to-yeast reversion, as confirmed by reduced hyphal scores. While additional quantification methods were considered to validate this outcome (eg, colony-forming units from vaginal tissue homogenate, fungal gene expression by quantitative polymerase chain reaction), previous studies have demonstrated comparable fungal loads by either method [12, 39]. The 24-hour delay in PMN reduction may reflect the time required for clearing the initial wave of PMN migration and/or diminished inflammatory triggers associated with hyphae-to-yeast transition.

Considering the multiple factors that contribute to the robustness of the mouse model favoring hyphal growth and chronic inflammation (eg, neutral pH, pseudoestrus conditions, and biofilm formation), any appreciable positive effects in mice could translate into substantial clinical impact for women. Indeed, such factors likely contributed to a rather modest outcome in mice compared with the clinical observations. PMNs were reduced only transiently at best in these mice and were never eliminated completely, supported by a lack of significant changes in levels of vaginal proinflammatory proteins. Another contributing factor was the limited cooling capacity of the magnetic rods (>29°C) compared with the Vlisse device containing a hydrogel (<25°C). Finally, the small sample size (3 mice per group) reduced the statistical power of this particular study yet was adequate to demonstrate evidence of no tissue damage from the cooling regimen. Although these animal experiments did not extend to later time points as in the clinical trial, an experiment with a 5-day treatment and 12-day observation showed similar trends through 48 hours after treatment, albeit with variable results over the subsequent days.

Despite the limitations and challenges with both the mouse model and the pilot trials, the results herein provide strong proof of principle for the intravaginal cooling approach to relieve VVC/RVVC symptoms, leading to clinical cure devoid of acute relapses. These findings justify further clinical trials to fully assess the potential of the Vlisse device as an effective treatment alternative for VVC/RVVC.

## Supplementary Material

jiaf028_Supplementary_Data

## Supplementary Data

Supplementary materials are available at *The Journal of Infectious Diseases* online (http://jid.oxfordjournals.org/). Supplementary materials consist of data provided by the author that are published to benefit the reader. The posted materials are not copyedited. The contents of all supplementary data are the sole responsibility of the authors. Questions or messages regarding errors should be addressed to the author.

## Notes

***Acknowledgments.*** The authors thank Keith Aqua, MD, at Atlantic Clinical Research Collaborative for valuable discussions regarding observations from investigator-initiated clinical trials. They also thank the study participants for their contributions to the pilot clinical trial and the vivarium staff for their assistance with animal care.

***Author contributions.*** K. L. and P. L. F. conceived and designed experiments and acquired funding. K. L. and M. S. enrolled study participants. J. Y. performed experiments. M. S. performed clinical examinations. J. Y., K. L., and P. L. F. analyzed data and wrote the manuscript. K. L. and M. C. N. provided materials. All authors reviewed and approved the final version of the manuscript.

***Financial support.*** This work was supported by the Carl Baldridge Endowed Chair fund through the LSU Health Foundation (P. L. F.), Coologics, Inc., (K. L.), and the National Institutes of Health via the National Institute of General Medical Sciences (NIGMS), which funds the Louisiana Clinical and Translational Science Center (grant U54 GM104940).
