# Supplementary material for: Use of Intravaginal Cooling to Provide Symptom Relief in Women With Vulvovaginal Candidiasis and Reduce Immunopathology in an Accompanying Mouse Model
Source: J Infect Dis. 2025 Jan 13;231(4):e813–21. doi: 10.1093/infdis/jiaf028 (PMC11998563; doi:10.1093/infdis/jiaf028)
Supplement: jiaf028_Supplementary_Data [file jiaf028_supplementary_data.zip › JID_81151_supplemental_table_1.docx]

**Table S1. Eligibility/exclusion criteria for enrollment.**

| Eligibility | Women between 18-55 years of age, or less than five years post last menses. | |
| --- | --- | --- |
|  | Patient with diagnosis for VVC via the following methods,   - history of VVC/RVVC episodes - pelvic examination - presence of *C. albicans* in vaginal secretions detected by yeast cultures - presence of *C. albicans* detected by wet mount microscopy/10% KOH - vaginal pH >4.5 | |
|  | Presence of at least one vulvovaginal sign at baseline.   - Erythema - Edema - Discharge | Scored 0-4   1. None 2. Minimal 3. Mild 4. Moderate 5. Severe |
|  | Presence of at least one vulvovaginal symptom at baseline.   - Itching - Burning - Pain - Irritation |  |
|  | Indication of moderate to severe VVC, defined by a composite vulvovaginal signs and symptoms score | Total score of ≥7 |
| Exclusion | Pregnant or nursing | |
|  | Diabetes mellitus, unless well-controlled | |
|  | Use of systemic, topical (applied to the vulva) or vaginal antibiotics, antifungals, or anti-trichomonas within seven days prior to randomization | |
|  | Use of any systemic corticosteroid, immunosuppressive, or immune-stimulating drug within three months prior to randomization | |
|  | Presence of concomitant genital infection (e.g., Trichomonas vaginalis, Chlamydia trachomatis, Neisseria gonorrhoeae, Herpes simplex virus). | |
|  | Presence of another vaginal or vulvar condition that would confound the interpretation of the clinical response. (e.g., dermatosis) | |
|  | History of an allergy or sensitivity to the device materials or related compounds | |
|  | Symptomatic vulvar or vaginal condyloma | |
|  | History of vulvodynia, vestibulitis, vaginismus, radiation-induced vaginitis, or postmenopausal atrophy | |
|  | Absence of menses for more than one year | |
|  | Immunocompromised states such as HIV/AIDS | |
|  | Chronic or recent steroid use | |
|  | Diminished mental capacity | |
|  | Unable or unwillingness to use tampons in the past | |
