# Supplementary material for: Use of Intravaginal Cooling to Provide Symptom Relief in Women With Vulvovaginal Candidiasis and Reduce Immunopathology in an Accompanying Mouse Model
Source: J Infect Dis. 2025 Jan 13;231(4):e813–21. doi: 10.1093/infdis/jiaf028 (PMC11998563; doi:10.1093/infdis/jiaf028)
Supplement: jiaf028_Supplementary_Data [file jiaf028_supplementary_data.zip › JID_81151_supplemental_text.docx]

**Supplemental materials**

**Methods**

**Intravaginal temperature measurements in women.** Temperature of the vaginal cavity during cooling treatment was measured using a digital temperature sensor installed with a stainless-steel probe (6 mm in diameter, 3.6 mm in length, and a precision of ± 0.5°C; MOCREO). The probe was securely attached to the intravaginal cooling device using sterile tape, and the assembly was stored at -20C for at least 2 h. Before use, the device was briefly rinsed under running tap water for 3 sec to moisten the surface and inserted into the vagina for 30 min or until the temperature readout reaches baseline vaginal temperature. Temperature measurements were recorded at 2-5 min intervals over the treatment period.

**Intravaginal temperature measurements in mice.** Temperature of the vaginal cavity was measured using a digital thermometer installed with a 19-mm-length rectal probe with a precision of ±0.1°C (ThermoWorks). At designated time points, mice were briefly restrained, and the probe was inserted into the vagina. Temperature measurements were taken at baseline and then repeated at each insertion at 10-min intervals for 40 min. Temperatures were recorded longitudinally for each animal.

**Lactose dehydrogenase assay.** Levels of LDH release in vaginal lavage fluids from mice treated with pre-cooled rods, pre-warmed rods or left untreated were measured as a marker of tissue damage. Lavage samples were diluted in PBS and analyzed for LDH activity using the CytoTox 96 non-radioactive cytotoxicity assay (Promega) per manufacturer’s instructions.

**Figure legends**

**Figure S1. Intravaginal cooling device for clinical use in women.** A tampon-shaped vaginal cooling device was designed by Coologics. The out shell of the device is composed of a medical-grade ethyl vinyl acetate material and filled with a proprietary hydrogel designed to sustain cold temperatures for a minimum of 30 min. One end of the device features a grip hole for secure insertion and removal by the user. Scale bar indicates 1 cm.

**Figure S2. Magnetic micro stir rod utilized for intravaginal cooling treatment in mice.** The magnetic rods were coated with smooth layers of an inert, FDA-grade polytetrafluoroethylene (PTFE) polymer. Prior to use, each magnetic rod was sterilized and pre-cooled to -20°C or pre-warmed to 35°C as a control. Rods were securely positioned along the full length of the vagina using fine-tipped forceps. Scale bar indicates 1 cm.

**Figure S3. Temperature measurements of the vaginal cavity during cooling treatment. (A)** Three healthy women self-administered intravaginal cooking treatment using the Vlisse device pre-cooled at -20°C and installed with a digital temperature sensor. Vaginal temperature readings were recorded at baseline and successive intervals of 2-5 min for a total duration of 30 min or until the readout reaches the baseline temperature. Dot lines indicate the temperature shifts per subject. **(B)** Mice were administered estrogen (0.1 mg/100 µl sesame oil) subcutaneously 3 days prior to the initial cooling treatment. Sterile magnetic micro rods, pre-cooled at -20 °C or pre-warmed at 35 °C, were placed into the vaginal cavity and replaced with fresh pre-warmed or pre-cooled rods every 10 min, for a total duration of 40-min. Vaginal temperature readings were recorded at baseline and after each rod application using a rodent rectal thermometer. After the treatment period, vaginal temperature was monitored for an additional 10 min. Dot lines represent group means with error bars indicating the mean ± SEM computed from independent replicates of two unique sets of animals examined longitudinally with 2-6 animals/group. **(C)** Vaginal lavage fluid was collected from mice treated with pre-cooled, pre-warmed, or left untreated. Levels of tissue damage were assessed by measuring LDH release using a non-radioactive cytotoxicity assay. Bars and error bars represent the group mean ± SEM of OD values computed from independent replicates of two unique sets of 2-4 animals/group. LDH levels in lavage fluid from *C. albicans* inoculated mice at 4 days post-inoculation were included as reference values for epithelial damage. Data were analyzed by using one-way ANOVA comparing across the subjects (A), two-way ANOVA with repeated measures comparing the treatment groups followed by Bonferroni posttests at each time point (B). or one-way ANOVA comparing the treatment groups (C). **, p < 0.01; ***, p < 0.001; ns, not significant.
