# Supplementary figures and images for: Use of Intravaginal Cooling to Provide Symptom Relief in Women With Vulvovaginal Candidiasis and Reduce Immunopathology in an Accompanying Mouse Model
Source: J Infect Dis. 2025 Jan 13;231(4):e813–21. doi: 10.1093/infdis/jiaf028 (PMC11998563; doi:10.1093/infdis/jiaf028)

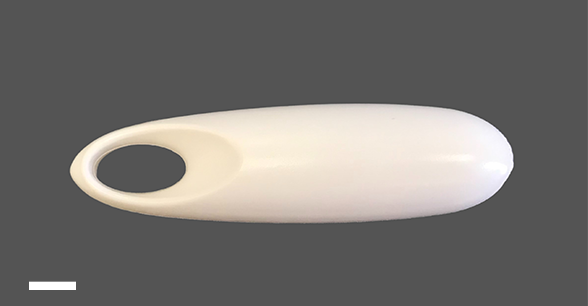

Supplement: jiaf028_Supplementary_Data [file jiaf028_supplementary_data.zip › Fig S1.tif]

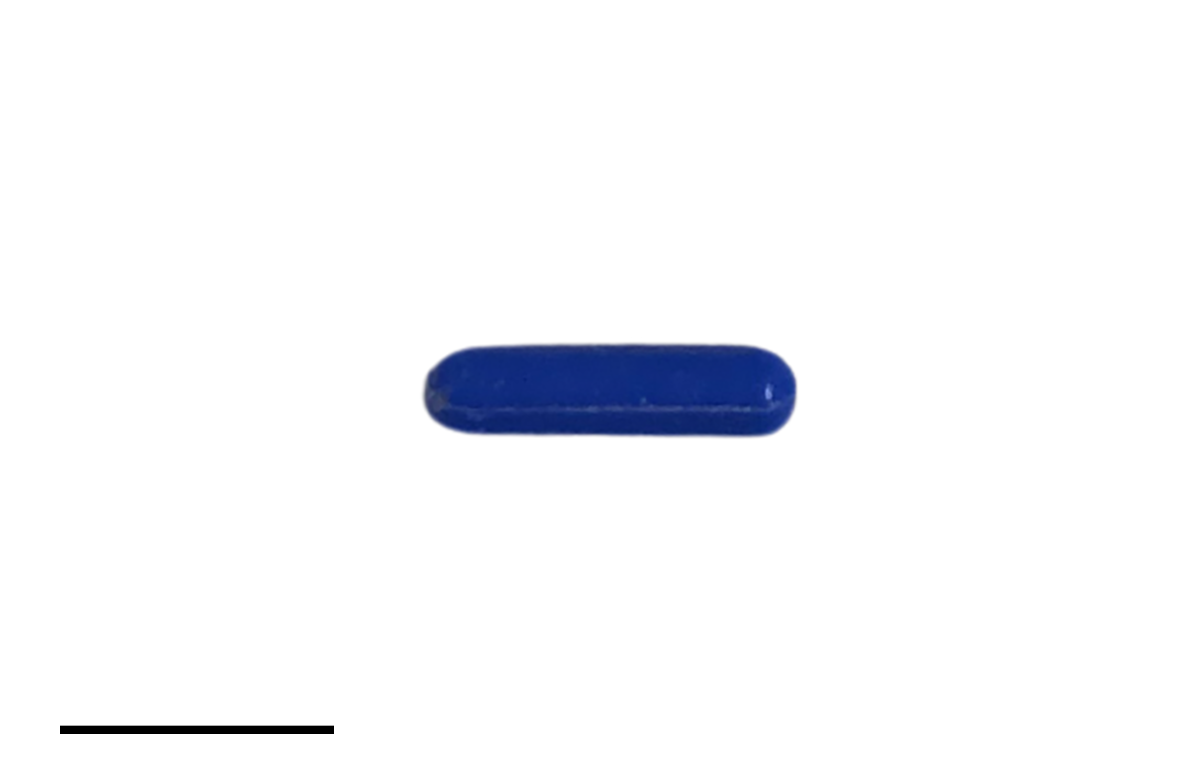

Supplement: jiaf028_Supplementary_Data [file jiaf028_supplementary_data.zip › Fig S2.tif]

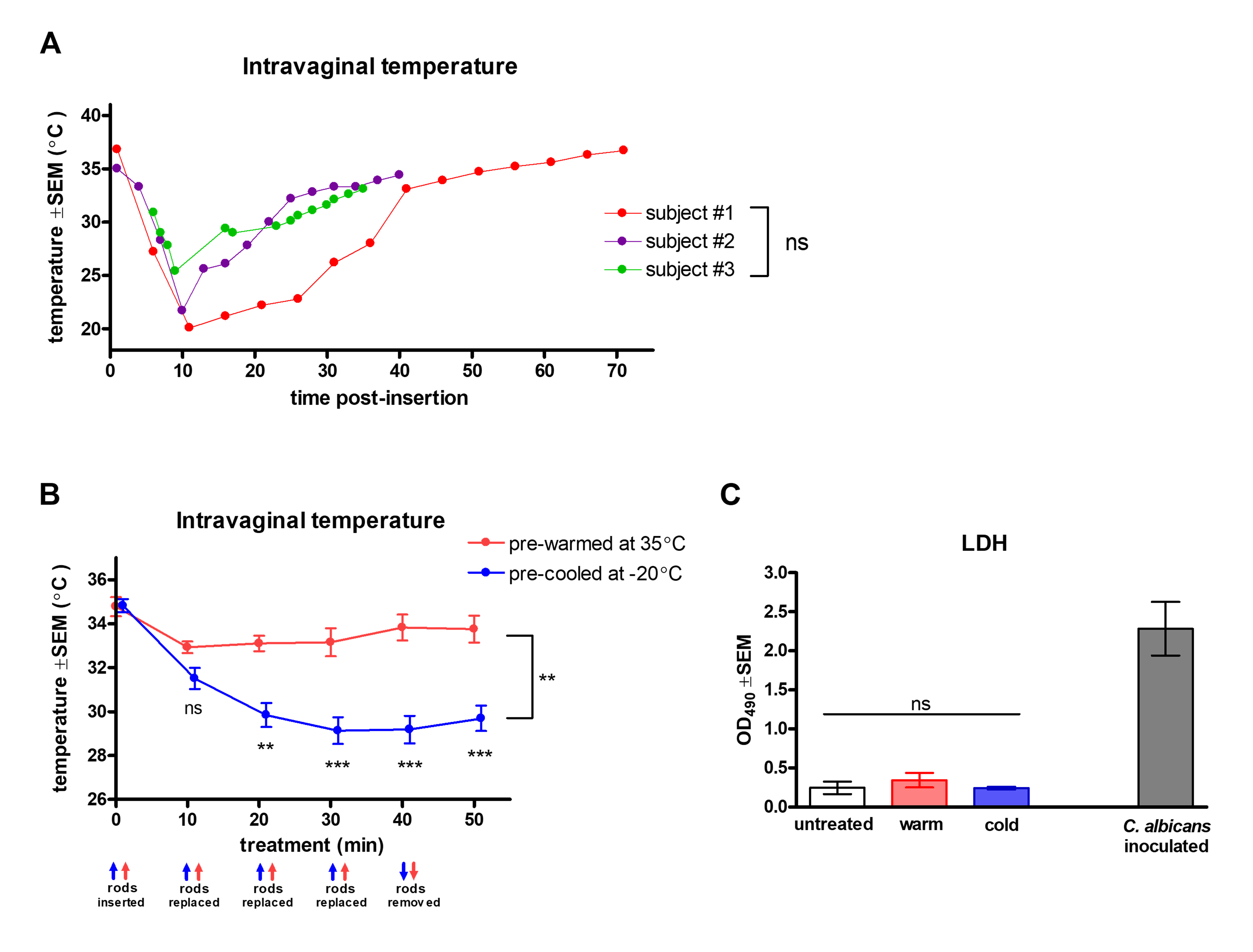

Supplement: jiaf028_Supplementary_Data [file jiaf028_supplementary_data.zip › Fig_S3.tif]
